# Designing a first-aid kit for use in commercial and fishing vessels based on possible types of injuries 

**Published:** 2019-07

**Authors:** Mohammad Nobakht

**Affiliations:** ^*a*^Marine Medicine Research Center, Baqiyatallah University of Medical Sciences, Tehran, Iran.

## Abstract

**Background:**

One of the major shortcomings in light and semi-heavy ships, such as fishing vessels, is the lack of first aid packages appropriate to the ship's mission and the type of injuries that may be caused by the crew. Therefore, a special bag should be designed to fit the folding stretcher and can carry medications and relief supplies.

**Methods:**

The hypotheses of this study included contents of the bag, its material, color, size, and weight, and the most importantly providing a component for facilitating in carrying. During the long-term studies, we designed a rescue backpack with special features that could be carried with the crew in any circumstances. The most important necessity for doing such a study was the need for rescue bags with proportionate size in light and semi-heavy floating vessels which can easily be placed in them and met all relief and rescue needs.

**Results:**

Each floating vessel may face various kinds of damages based on its mission type. These require the contents of the ship's rescue kit to be modified in accordance with the type of mission and the type of injury involved. Although the type of possible injuries can be largely similar, some vessels should have more complete qualities or proprietary equipment due to their type of structure and type of mission.

**Conclusions:**

Today, even systems equipped with numerous supplies for rapid rescue and transportation such as NATO rescue systems still provide their medical staff with high quality equipment. In other words, NATO troops, which benefit from a variety of protective and anti-bullet covers, as well as diverse and fast-moving vehicles for rescue and transportation, still have special emphasis on relief efforts at the scene of the incident. Therefore, it is desirable to pay special attention to the rescue and relief in both military and civilian floating vessels.

**Keywords:**

Rescue bag, Light and Semi-heavy Floating Vessels, Possible injuries

